# Importance-Performance Analysis of Clinical Forensic Services Quality at Bhayangkara Hospital Pekanbaru, Indonesia

**DOI:** 10.21315/mjms2024.31.1.9

**Published:** 2024-02-28

**Authors:** Dedi Afandi, Merita Arini

**Affiliations:** 1 Forensic Medicine and Medicolegal Department, Faculty of Medicine, Universitas Riau, Pekanbaru, Indonesia; 2 Master of Hospital Administration Study Program, Postgraduate Program, Universitas Muhammadiyah Yogyakarta, Yogyakarta, Indonesia

**Keywords:** forensic clinic, importance-performance analysis, service quality

## Abstract

**Background:**

Service quality improvement efforts must focus on the utilisation of resources for continuous quality improvement. The importance-performance analysis (IPA) method is useful in finding service quality items that require corrective action. This study implemented the IPA method to obtain items that should be prioritised in improving the quality of a hospital’s clinical forensic services.

**Methods:**

A cross-sectional study of 284 clinical forensic patients at Bhayangkara Hospital, Pekanbaru, Indonesia was conducted. Self-administered and paper-based questionnaires, specifically the modified service quality (SERVQUAL)-based questionnaire, were used as the study instruments. Twenty-two service quality items were used as indicators and they were divided into five dimensions of service quality: i) reliability, ii) responsiveness, iii) assurance, iv) empathy and v) tangibility. The data were analysed using the IPA method.

**Results:**

The results showed that only seven items had a gap and a level of conformity that met the expectations of clinical forensic patients. Improvements in service quality should prioritise four items: i) providing a more private examination room for clinical forensic patients, ii) improving healthcare workers’ understanding of patient needs, iii) improving the readiness of healthcare workers to conduct examinations and iv) enhancing the clarity of information about examination procedures.

**Conclusion:**

The IPA method yielded several high-priority items that need to be improved; therefore, the hospital must focus on improving the quality of clinical forensic services.

## Introduction

Measurements of service quality are done to evaluate and determine the quality of hospital services. Continuous and in-depth measurements can determine service quality more precisely and the results can be used to improve patient satisfaction (1–3). Patient satisfaction is strongly influenced by patients’ perceptions of the services received. Patients will feel satisfied if they perceive that they have obtained a quality of care that exceeds their expectations (4, 5). Patient satisfaction with health services is demonstrative of their overall quality; therefore, measuring service quality can help improve patient satisfaction (5, 6).

Service quality can be measured using various methods and instruments but a questionnaire is most often used. Other methods include interviews, focus group discussions and investigations into patient complaints and claims (1, 3). Previous studies on the variables affecting service quality and patient satisfaction have revealed various challenges, emphasising the need for continuous attention to issues affecting service quality and to ensuring that hospital managers are informed about potential issues for further problem-solving steps (7, 8).

However, the research on service quality has several weaknesses, particularly in the measuring instruments used. Measurements using service quality (SERVQUAL) instrument enable only an attribute gap analysis and cannot prioritise items or dimensions that must be improved. Furthermore, research has shown that hospital performance does not meet patient expectations based on various quality dimensions (9). Therefore, it is necessary to focus on improvement models that benefit patients and consider their interests in a hospital as a place where they can obtain health services (8, 10). For this reason, efforts have been made to improve service quality by using importance-performance analysis (IPA) analysis (8, 9).

Martilla and James (18) introduced the first quadrant analysis to determine the correlation between perceptions and priorities for improving products or service quality. This method is known as the IPA method. The IPA method is a reliable analytical tool for practitioners and academics, and it finds attributes that require corrective action. IPA allows hospital management and other health services to understand patient perceptions and expectations (8, 11, 12).

Clinical forensic services are one of the services hospitals must provide. Forensic medical examinations are carried out to determine the type of wound, type of violence and degree of violence perpetrated on patients who are victims of violence. The result of clinical forensic services is a medicolegal certificate (13). Measuring the level of patient satisfaction with service quality or using other methods to survey clinical forensic patients, has never been done. At Bhayangkara Hospital, Pekanbaru, patients often compare the results of forensic medical examinations with results from other hospitals. However, no in-depth investigation into why this happens has been conducted. Therefore, it is unclear why patients may be dissatisfied with the examination results from Bhayangkara Hospital, Pekanbaru. Thus, this study implemented the IPA method to obtain service quality attributes that require improvement at Bhayangkara Hospital, Pekanbaru.

## Methods

### Study Design and Participant Characteristics

A cross-sectional study design was adopted. This study was conducted in the Emergency Department of Bhayangkara Hospital, Pekanbaru from April 2022 to October 2022. The study population included all patients who registered as clinical forensic patients in the Emergency Unit (ER) of Bhayangkara Hospital, with the inclusion criteria being that patients were victims of violence with a maximum degree of moderate injury, aged over 18 years old, in a state of consciousness and able to read and write. The criteria for the degree of injury were based on the laws and regulations in force in Indonesia. The determination of the degree of injury was carried out using the results of a physical examination, the medical treatment performed and the results of supporting examinations (e.g. X-ray, CT scans and laboratory results). Furthermore, expert examination by a specialist in forensic medicine was carried out to relate the patient’s condition to the regulations. Moderate injury was defined as that presenting on a patient who was a victim of violence with injuries that required medical treatment but did not fulfil the provisions of Article 90 of the Criminal Code in Indonesia. Severe injuries were those that met the provisions of Article 90 of the Criminal Code in Indonesia. Serious physical injuries were defined as illnesses or injuries that do not leave any prospect of a complete recovery or through which life endangerment exists; that result in continuous incompetence in performing official and professional activities; that contribute to the loss of the use of a sensory organ; and that involve mutilation, paralysis, disturbance of intellectual capabilities that lasts for more than 4 weeks or removal or death of the womb of a woman (14).

The minimum sample size was calculated using the formula for estimating the proportion of samples with a *Z* of 1.96, P of 0.76 from the results of previous studies (15) and a precision value of 5%. The formula for calculating the minimum sample size is shown below:


$$\begin{array}{l} \text{Minimum sample size}=\frac{Z^{2}\text{P}(1-\text{P})}{d^{2}}\\ =\frac{{\left(1.96\right)}^{2}\times 0.76\times (1-0.76)}{{\left(0.05\right)}^{2}}=281 \end{array}$$

Thus, the calculated minimum sample size was 281 respondents. The sampling technique used was a consecutive sampling approach.

### Study Instrument

Self-administered and paper-based questionnaires, specifically the modified SERVQUAL-based questionnaire, were used as the study instruments. A total of 22 service quality items were used as indicators, and these were divided into five dimensions of service quality: i) reliability, ii) responsiveness, iii) assurance, iv) empathy and v) tangible (Table 2). In addition, the measurement scale was modified from the original version—from a 7-point Likert scale to a 5-point Likert scale ranging from 1 (strongly disagree) to 5 (strongly agree) in the Perceived (P) section of the study and from 1 (very unimportant) to 5 (very important) in the Expectation (E) section. In the P section, patients gave a score on the quality of services they received, while in the E section, patients gave a score on how important the service was to them.

This questionnaire had sufficient validity based on the recommendations set forth by the Standards for Educational and Psychological Testing from the American Educational Research Association (AERA), the American Psychological Association (APA) and the National Council on Measurement in Education (1999) (16, 17). Written permission to modify the questionnaire was obtained from the developer of the original version. The questionnaire has validity based on evidence pertaining to its content, the response process and some part of its internal structure, specifically the item-to-total correlation and reliability. The instrument was thus considered reliable, with a Cronbach’s alpha score of 0.956 and all items had a total correlation coefficient (*r*) greater than the *r*-table (0.361), which ranged from 0.557 to 0.827 (14).

### Data Analysis

The data were analysed using the IPA method. The study steps included a gap analysis, the calculation of the level of conformity and the determination of the IPA matrix. A gap analysis was conducted by subtracting the E mean scores from the P mean scores to obtain the P–E gap. The conformity level (P/E) was determined by comparing the P mean scores with the E mean scores.

The IPA matrix in the form of an IPA Cartesian diagram was divided into four quadrants, which were bounded by two axes that intersect perpendicularly at point (*x*, *y*), where the P means were the *x*-axis and the E means were on the *y*-axis. The items could then be analysed based on resource allocation priorities (8, 12, 18). The IPA matrix was created and analysed using the Statistical Package for Social Sciences (SPSS) version 23.0 (USA).

Each quadrant of the IPA matrix was interpreted as follows: i) Quadrant I: the ‘concentrate here’ area, is the most crucial area in the IPA Matrix. Items and dimensions in this quadrant are considered underperforming, thus representing the product’s main weaknesses and threats to its competitiveness. Therefore, this item or dimension has the highest priority in terms of investment; ii) Quadrant II: ‘keep up the good work’, represents the essential strengths and potential competitive advantages of a service or product. It is assumed that the items and dimensions in this quadrant are performing well and require continued investment; iii) Quadrant III: features items and dimensions included in the ‘low priority’ area that do not perform very well but are considered relatively unimportant to customers; therefore, managers should not be concerned with this quadrant. These items and dimensions represent minor flaws and poor performance but do not seriously impact service quality and iv) Quadrant IV: the ‘possible overkill’ area, contains items/dimensions that are not very important to the customer but perform strongly, indicating the possible wasting of limited resources that are being used inefficiently and could be reallocated elsewhere (9, 18).

## Results

At the end of the data collection process, as many as 284 clinical forensic patients were willing to participate in the entire series of studies and complete the questionnaire. The characteristics of the research respondents are shown in Table 1.

More than half of the respondents were male (53.3%) and the rest were female (46.5%). The median age was 29 years ols, with the lowest age being 18 years old and the highest being 75 years old. The age group of 21–30-year-olds was the largest both overall (41.2%) and among men (43.4%) and women (38.6%). The majority (165; 58.1%) had a high school education, which was further replicated across both males (63.2%) and females (52.3%). Being self-employed was the most common type of work (33.5%), followed by not working (22.2%) and being a private employee (19%). As many as 51.1% of the respondents were married.

The IPA was carried out in stages. This began with the gap analysis. Then, the level of conformity was calculated and a Cartesian diagram was created. The results of the gap analysis and the level of service quality conformity are shown in Table 2. The gap rank was carried out for each item and service quality dimension.

From the calculations, the smallest to the largest gap was between −0.680 and 0.102. There were seven items with positive gap values: R1, R3, R4, R5, A4, E5 and T1. The calculation results showed the gap rank for each item; the lowest-ranking item was T2 and the highest was R4. Based on the dimensions of service quality, a positive gap was found only in the reliability dimension (X1) and the other four dimensions had a negative gap. The gap ranks from the lowest to the highest were responsiveness (X2), tangibility (X5), empathy (X4), assurance (X3) and reliability (X1). Overall, the service quality gap analysis yielded a value of −0.176.

The conformity of each item and the dimensions of service quality are presented in Table 2. The level is shown as a percentage, where 100% indicated that the respondents were fully satisfied with the service they received.

Only seven items achieved a service conformity level above 100%, meaning that the patients were satisfied and as many as 15 items were below 100% (not satisfied). Almost all the items achieved a percentage score of more than 90%, except for item T2, which obtained only 86.1%. Of the five service quality dimensions, only the reliability dimension (X1) had conformity above 100%, which means that the respondents were satisfied with this dimension. The mean value of the overall conformity level was 96.6% (not satisfied).

**Figure 1:**
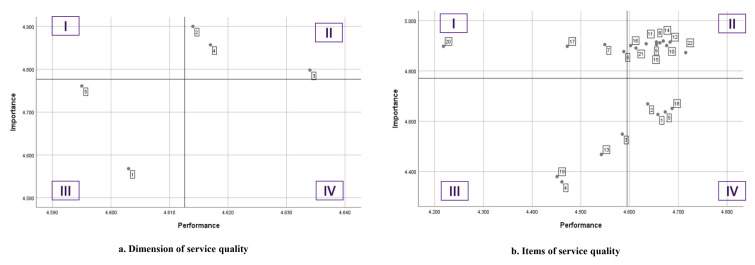
Cartesian diagram of IPA

The IPA matrix in the form of a Cartesian diagram is shown in Figure 1. The distribution of each dimension in the diagram is shown in Figures 1(a) and (b).

The Cartesian diagram in Figure 1(a) shows that the service quality dimensions were spread out in Quadrants II and III. Quadrant II shows that the dimensions of service quality that were considered good and need to be maintained were the dimensions of responsiveness (X2), empathy (X4) and assurance (X3). Tangible dimensions (X5) and reliability (X1) were in Quadrant III, and hospitals should consider these worthies of improvement but not a high priority.

In Figure 1(b), the items are distributed in all four quadrants and can be interpreted as follows: i) items Rs1, Rs2, E4 and T2 are in Quadrant I, which means that they require immediate attention for service improvement; ii) items Rs3, Rs4, A1, A2, A3, E1, E2, E3, T3 and T4 are in Quadrant II, which means that the services provided by the hospital have been good and are considered important by the respondents; iii) items R3, R4, A4 and T1 are in Quadrant III, which means that hospitals should consider these items worthy of improvement but not a high priority and iv) items R1, R2, R5 and E5 are distributed in Quadrant IV, which means that the hospital is performing well but these items are not a top priority.

## Discussion

The IPA method is widely used to analyse the different factors and dimensions of service quality to prioritise them for improvement (9, 11, 18, 19). In line with this, our study implemented the IPA method to obtain priority items and dimensions to improve clinical forensic services at Bhayangkara Hospital, Pekanbaru.

Respondents were asked to rate the level of importance and performance of the hospital. The average values of the levels of importance and performance were analysed using the IPA matrix, where the *x*-axis represented performance and the *y*-axis represented interest. The degree of conformity was the result of comparing the performance score with the importance score. This level of conformity was used to determine the order of priority in improving the factors that affect customer satisfaction (9, 17). This study used gap analysis, calculated conformity levels and constructed the IPA matrix.

### Gap Analysis

The gap in overall service quality was −0.176. This indicates that the quality of clinical forensic services at Bhayangkara Hospital, Pekanbaru did not meet patients’ expectations. This result aligns with the study conducted by Zarei et al. (20), who showed a negative gap value (−0.65) for overall service quality. Good service quality should have a positive gap value, which means that patient expectations were met (9, 15).

The results of this study are different from the results of a study conducted in Taiwan in 2020 that obtained a positive gap (0.070), which means that the service quality provided by the hospital met patient expectations (11). This is mainly due to differences in the facilities provided by the hospitals, the study populations, the study locations in the intensive care unit (ICU) and the type of service, specifically intensive therapy.

The results of the gap analysis of service quality are as follows. There was one dimension with a positive gap value, specifically reliability (X1) at 0.035 and the other four dimensions had a negative gap value. Responsiveness (X2) had a value of −0.286, tangibility (X5) −0.261, empathy (X4) −0.240 and assurance (X3) −0.165. This means that only the perceived reliability dimension fulfilled patient expectations. A study conducted in Iran in 2020 obtained negative gap scores for all quality dimensions. Meanwhile, a study conducted in Taiwan in 2020 yielded more dimensions with positive gap values, specifically tangibility (0.32), empathy (0.15), and responsiveness (0.01) (11, 20). This difference is likely because of the different indicators the studies used to assess each dimension, the different study populations and the different types of patient services.

These results indicated that only 7 of the 22 items fulfilled patient expectations regarding service quality. The item with the best performance was forensic examination, which was always carried out immediately. This result is in line with previous studies’ findings that waiting time affects the assessment of service quality by patients and that short waiting times increase patient satisfaction (8, 21, 22). Richter et al. (23) found that it is not enough for a service to be available 24 h a day to increase patient satisfaction. It is more important that patients receive medical treatment immediately. Seven items met the criteria for good service quality to be maintained and continuously improved. Skilled and experienced healthcare workers, thorough identity and wound checks and prompt examinations will guarantee patient satisfaction and trust in the hospital.

A very negative gap value was found in the assessment of the factor ‘the examination room that maintains privacy’ (−0.680). This means that the clinical forensic examination rooms at Bhayangkara Hospital have not fulfilled patient expectations. Privacy is an important issue that hospitals must address, particularly for victims of sexual crimes who require an examination of the anogenital area. Studies have shown that privacy is closely related to patient satisfaction. There is a significant difference in a patient’s perceptions of privacy for a room that is not private and for a more private room (24). The layout of the examination room also affects patient satisfaction. Patients are more satisfied in a room within reach or under the close supervision of doctors and healthcare workers (25).

### Conformity Level

Overall, the level of conformity of clinical forensic service quality at Bhayangkara Hospital was 96.6%, which means that clinical forensic patients did not find the services to be satisfactory. The dimensions of service quality that had a satisfactory level of conformity were reliability (X1) at 100.8%, but the other four dimensions showed that patients were not satisfied, with assurance (X3) at 96.6%, tangibility (X5) at 96.5%, empathy (X4) at 95.1% and responsiveness (X2) at 94.2%. The higher the satisfaction percentage, the more satisfied the patient will be (10). Therefore, a level of conformity above 100% indicates that the patient is satisfied, while a level below 100% demonstrates that the patient is not satisfied (9, 10).

Purba et al. (10) found that the overall level of conformity in service quality was lower than the results of this study at 62.44%, which means that patients were slightly satisfied with service performance. This discrepancy was due to population differences and the indicators used in the measurements.

### IPA Matrix

The distribution of service quality dimensions and the items in each quadrant were obtained. There was no distribution of service quality dimensions in Quadrants I and IV. In Quadrant II (‘keep up the good work’), there were dimensions of responsiveness (X2), empathy (X4) and assurance (X3), meaning these dimensions were performing well; therefore, Bhayangkara Hospital needs to maintain the performance of these dimensions. The tangibility (X5) and reliability (X1) dimensions were in Quadrant III (low priority), which means that Bhayangkara Hospital should consider improving the performance of these dimensions, even though they are not a high priority.

This study obtained the distribution of items in the four quadrants. The highest number of items was distributed in Quadrant II (‘keep up the good work’), which included 10 items, meaning that almost half of the service quality items were performing well and should be maintained. In Quadrants I, III and IV, there were four items.

The priority items for improvement were four items in Quadrant I (‘concentrate here’), which are ‘the examination room maintains privacy’ (T2), ‘healthcare workers understand patient needs’ (E4), ‘healthcare workers are wide awake to carry out forensic examinations’ (Rs2) and ‘healthcare workers provide information about forensic examination procedures clearly’ (Rs1). This was also supported by the gap analysis results for each of these items, all of which yielded a negative gap value with a conformity level below 100%.

Several studies have shown the importance of maintaining patient privacy, which can be achieved by providing an examination room that is closed and separated from other rooms so that patients feel comfortable during examinations (24, 26). In the case of sexual crimes, the World Health Organization (WHO) (27) issued a standard for clinical forensic examination rooms, stating that they must be private, quiet, easily accessible, clean and have access to a toilet or latrine.

Kerbacher et al. (28) recommended that continuous training be carried out so that healthcare workers’ ability to manage clinical forensic patients meets patient expectations. An explanation of the examination procedure must be provided, and patient approval of the medical action must be obtained before a clinical forensic examination is carried out (29, 30). If this is performed correctly, the examination can run smoothly, thereby increasing patient satisfaction.

The non-tangible items that meet patient expectations and should be maintained by Bhayangkara Hospital, Pekanbaru are as follows: healthcare workers are willing to help in the forensic examination process (Rs3); healthcare workers are ready to respond to complaints, problems or questions (Rs4); healthcare workers introduce themselves before starting the examination (A1); healthcare workers provide a sense of comfort when the examination is carried out (A2); healthcare workers show respect during forensic examinations (A3); healthcare workers pay special attention to patients based on their cases (E1); healthcare workers listen carefully to complaints, chronology and events (E2) and healthcare workers serve with all their hearts (E3). These results indicate that non-tangible factors related to healthcare workers’ ability significantly increased the satisfaction of clinical forensic patients examined and treated at Bhayangkara Hospital, Pekanbaru.

Tangible items that were performing well and need to be maintained are as follows: healthcare workers have a neat appearance (T3) and the forensic examination equipment looks clean (T4). Previous studies have proven that tangibility is a service quality dimension that affects patient satisfaction (31–33).

Studies that applied the IPA method have obtained varying results for the dimensions and items that should be prioritised for improvement. Lu et al. (11) reported only one item that needed priority improvement. Purba et al. (10) reported eight items that required priority improvement. Zarei et al. (20) reported the tangibility dimension and four items requiring improvement. This proves that the IPA method helps hospital management improve service quality and patient satisfaction rates based on a priority scale.

The main limitation of this study is that we cannot further investigate the extent to which the items that were below patient expectations need to be improved. This issue can be addressed through further studies that adopt qualitative research methodologies using various data collection methods, such as focus group discussions, in-depth interviews or questionnaires with open-ended questions. This research was conducted on clinical forensic services that do not require payment, so there could have been information bias in respondents’ assessment of satisfaction and service quality compared to research carried out in a different hospital setting in another area with different socioeconomic and cultural conditions.

## Conclusion

Based on the IPA method, it can be concluded that only a small portion of the service quality indicators had a gap and a level of conformity that met the expectations of clinical forensic patients. Therefore, it is necessary to improve service quality by prioritising the provision of a more private examination room for clinical forensic patients, enhancing healthcare workers’ understanding of patient needs, enhancing the alertness of healthcare workers in carrying out examinations and enhancing the clarity of information about examination procedures. Furthermore, additional research using a qualitative approach is required to clarify items according to patients’ expectations in different research locations, especially regarding paid clinical forensic services.

## Figures and Tables

**Table 1:** Characteristics of the respondents (*N* = 284)

Variable	Gender, *n* (%)		Total *n* (%)

	Male	Female	
Age (years old), median (min–max)	30 (19–60)	28 (18–75)	29 (18–75)
18–20	12 (7.9)	22 (16.7)	34 (12)
21–30	66 (43.4)	51 (38.6)	117 (41.2)
31–40	43 (28.3)	32 (24.2)	75 (26.4)
41–50	22 (14.5)	21 (15.9)	43 (15.1)
51–60	9 (5.9)	5 (3.8)	14 (4.9)
> 60	0 (0)	1 (0.8)	1 (0.4)
Education			
Elementary	7 (4.6)	11 (8.3)	18 (6.3)
Medium	12 (7.9)	16 (12.1)	28 (9.9)
High School	96 (63.2)	69 (52.3)	165 (58.1)
Diploma/Undergraduate	35 (23)	35 (26.5)	70 (24.6)
Postgraduate	2 (1.3)	1 (0.8)	3 (1.1)
Work			
Unemployed	9 (5.9)	54 (40.9)	63 (22.2)
Student	6 (3.9)	4 (3)	10 (3.5)
Housewife	0 (0)	20 (7)	20 (7)
Government employee	9 (5.9)	3 (2.3)	12 (4.2)
Private employee	38 (25)	16 (12.1)	54 (19)
Irregular employee	18 (11.8)	4 (3)	22 (7.7)
Professional	0 (0)	5 (3.8)	5 (1.8)
Self-employed	70 (46.1)	25 (18.9)	95 (33.5)
Other	2 (1.3)	1 (0.8)	3 (1.1)
Marital status			
Unmarried	76 (50)	63 (47.7)	139 (48.9)
Married	76 (50)	69 (52.3)	145 (51.1)

Total	152 (53.3)	132 (46.5)	284 (100)

**Table 2:** Descriptive results of gap analysis and service conformity level

Dimension-Item^a^	Perception (P)		Expectation (E)		Gap analysis		Conformity	

	Total	Mean (SD)	Total	Mean (SD)	P-E^b^	Rank	P/E	%^c^
**Reliability (X1)**	**6,536**	**23.01 (1.32)**	**6,487**	**22.84 (1.32)**	**0.035**	**1**	**1.008**	**100.8**
Healthcare workers are skilled in conducting forensic examinations (R1)	1323	4.66 (0.47)	1314	4.63 (0.48)	0.032	5	1.007	100.7
Healthcare workers ask for complaints in depth (R2)	1317	4.64 (0.48)	1326	4.67 (0.47)	−0.032	6	0.993	99.3
The identity-check procedure is carried out carefully (R3)	1302	4.58 (0.49)	1292	4.55 (0.58)	0.035	4	1.008	100.8
The forensic examination process is carried out immediately (R4)	1267	4.46 (0.61)	1238	4.36 (0.59)	0.102	1	1.023	102.3
Healthcare workers examine wounds/trauma accurately (R5)	1327	4.67 (0.47)	1317	4.64 (0.51)	0.035	4	1.008	100.8
**Responsiveness (X2)**	**5,241**	**18.45 (1.20)**	**5,566**	**19.60 (0.84)**	−**0.286**	**5**	**0.942**	**94.2**
Health workers provide information about forensic examination procedures clearly (Rs1)	1303	4.59 (0.49)	1385	4.88 (0.35)	−0.289	14	0.941	94.1
Healthcare workers are wide awake to carry out forensic examinations (Rs2)	1292	4.55 (0.58)	1393	4.90 (0.32)	−0.356	16	0.927	92.7
Healthcare workers are willing to help in the forensic examination process (Rs3)	1324	4.66 (0.47)	1395	4.91 (0.31)	−0.250	10	0.949	94.7
Health workers are ready to respond to complaints/problems/questions (Rs4)	1322	4.65 (0.48)	1393	4.90 (0.34)	−0.250	10	0.949	94.9
**Assurance (X3)**	**5,264**	**18.54 (1.09)**	**5,451**	**19.19 (0.95)**	−**0.165**	**2**	**0.966**	**96.6**
Healthcare workers introduce themselves before starting the examination (A1)	1328	4.68 (0.47)	1392	4.90 (0.34)	−0.225	8	0.954	95.4
Healthcare workers provide a sense of comfort when the examination is carried out (A2)	1316	4.63 (0.51)	1394	4.91 (0.31)	−0.275	12	0.944	94.4
Healthcare workers show respect during forensic examinations (A3)	1330	4.68 (0.48)	1396	4.92 (0.31)	−0.232	9	0.953	95.3
Healthcare workers are experienced in handling forensic patients (A4)	1290	4.54 (0.51)	1269	4.47 (0.61)	0.074	2	1.017	101.7
**Empathy (X4)**	**6,556**	**23.08 (1.38)**	**6,897**	**24.29 (1.04)**	−**0.240**	**4**	**0.951**	**95.1**
Healthcare workers pay special attention to patients based on their cases (E1)	1326	4.67 (0.47)	1397	4.92 (0.33)	−0.250	10	0.949	94.9
Healthcare workers listen carefully to complaints/chronology/events (E2)	1322	4.65 (0.47)	1396	4.92 (0.30)	−0.261	11	0.947	94.7
Healthcare workers serve with all their hearts (E3)	1307	4.60 (0.49)	1392	4.90 (0.32)	−0.299	15	0.939	93.9
Healthcare workers understand the patient’s needs (E4)	1270	4.47 (0.61)	1391	4.90 (0.35)	−0.426	17	0.913	91.3
Healthcare workers are always available when needed (E5)	1331	4.69 (0.46)	1321	4.65 (0.48)	0.035	4	1.008	100.8
**Tangible (X5)**	**5,111**	**18 (1.27)**	**5,408**	**19.04 (0.89)**	−**0.261**	**3**	**0.965**	**96.5**
The examination room is clean (T1)	1264	4.45 (0.53)	1244	4.38 (0.60)	0.070	3	1.016	101.6
The examination room maintains privacy (T2)	1198	4.22 (0.62)	1391	4.90 (0.35)	−0.680	18	0.861	86.1
The healthcare workers have a neat appearance (T3)	1310	4.61 (0.49)	1389	4.89 (0.34)	−0.278	13	0.943	94.3
The forensic examination equipment looks clean (T4)	1339	4.71 (0.45)	1384	4.87 (0.38)	−0.158	7	0.967	96.7

**Service quality (X)**	**28,708**	**101.08 (4.62)**	**23,884**	**104.96 (3.34)**	−**0.176**		**0.966**	**96.6**

Notes:

a an original version in Bahasa Indonesia;

b P-E gap value equal to 0 means that there is no difference between performance and expectations; a P-E gap > 0 indicates that performance exceeds patient expectations and a P-E gap < 0 indicates that performance has not met patient expectations;

c The interpretation: > 100% of patients are satisfied and below 100% of patients are not satisfied
